# Increased proliferation of hepatic periportal ductal progenitor cells contributes to persistent hypermetabolism after trauma

**DOI:** 10.1111/jcmm.14845

**Published:** 2019-12-03

**Authors:** Li Diao, Yusef Yousuf, Saeid Amini‐Nik, Marc G. Jeschke

**Affiliations:** ^1^ Sunnybrook Research Institute Toronto ON Canada; ^2^ Division of Plastic Surgery Department of Surgery University of Toronto Toronto ON Canada; ^3^ Department of Laboratory Medicine and Pathobiology (LMP) University of Toronto Toronto ON Canada; ^4^ Department of Immunology University of Toronto Toronto ON Canada; ^5^ Ross Tilley Burn Centre Sunnybrook Health Sciences Centre Toronto ON Canada

**Keywords:** burns, hepatocytes, inflammation, lineage‐trace, liver regeneration, liver X receptor, metabolism, periportal ductal progenitor cell, stress response

## Abstract

Prolonged and persistent hypermetabolism and excessive inflammatory response after severe trauma is detrimental and associated with poor outcome. The predisposing pathology or signals mediating this complex response are essentially unknown. As the liver is the central organ mediating the systemic metabolic responses and considering that adult hepatic stem cells are on top of the hierarchy of cell differentiation and may pass epigenetic information to their progeny, we asked whether liver progenitor cells are activated, signal hypermetabolism upon post‐traumatic cellular stress responses, and pass this to differentiated progeny. We generated Sox9CreER^T2^: ROSA26 EYFP mice to lineage‐trace the periportal ductal progenitor cells (PDPCs) and verify the fate of these cells post‐burn. We observed increased proliferation of PDPCs and their progeny peaking around two weeks post‐burn, concomitant with the hepatomegaly and the cellular stress responses. We then sorted out PDPCs, PDPC‐derived hepatocytes and mature hepatocytes, compared their transcriptome and showed that PDPCs and their progeny present a significant up‐regulation in signalling pathways associated with inflammation and metabolic activation, contributing to persistent hypermetabolic and hyper‐inflammatory state. Furthermore, concomitant down‐regulation of LXR signalling in PDPCs and their progeny implicates the therapeutic potential of early and short‐term administration of LXR agonists in ameliorating such persistent hypermetabolism.

## 1 INTRODUCTION

Severe trauma such as major burn injury is always accompanied by acute perturbation of homeostasis and substantial stress responses with profound metabolic alterations, termed the hypermetabolic stress response.^1^, ^2^, ^3^, ^4^ Multiple clinical studies demonstrated that the hypermetabolic response after major burn injury is profound, prolonged and includes massive pro‐inflammation, but more importantly persists for years after the insult thereby contributing to significant morbidity and mortality.^5^, ^6^ The underlying mechanisms of how extensive burn injury leads to prolonged hypermetabolism are still not known. As the liver is the functional hub of immunologic, metabolic, inflammatory and acute phase responses, hepatic response to thermal injury is important in the development of post‐burn pathology.^7^ Considering the ability and plasticity of continuous self‐regeneration of the liver,^8^ we speculated and sought to test that pathological changes in hepatocytes’ proliferation and liver regeneration under stress conditions contribute to such prolonged hyper‐inflammatory and hypermetabolic states.

Although it has been well demonstrated that the liver has the capacity to regenerate and up to 2/3 of the loss of the liver parenchyma can be recovered by regeneration without jeopardizing the viability of the entire organism,^9^, ^10^ there are still controversies on how such a regeneration happens including whether there is single or multiple sources of stem cells, what the triggers of the liver regeneration are, and how the liver regeneration is regulated.^11^, ^12^, ^13^ As the portal triads are where the facultative regeneration of hepatic parenchyma occurs under liver damage and stress conditions,^14^ in line with the existing streaming liver theory 15, 16 that the regeneration and maturation of hepatocytes start from the portal venule, proceed across the liver plates and end with clearance in the central venule, we suggested that liver regeneration under profound stress condition would be dominated by proliferation and differentiation of periportal ductal progenitor cells (PDPC) which are bi‐potential progenitor cells that can give rise to either hepatocytes or cholangiocytes,^17^ whereas liver regeneration under physiological or mild stressful conditions was dominated by self‐duplication of mature hepatocytes.^12^ We further speculated that those hepatocytes regenerated under significant stress conditions after major burn injury might possess aberrant and persistent inflammatory and/or hypermetabolic profiles and thus contribute to prolonged pro‐inflammatory states and hypermetabolism that are commonly seen in major burned patients.^5^, ^6^

## 2 MATERIALS AND METHODS

### 2.1 Animal studies

Animal experiments were approved by the Animal Care and Use Committee of Sunnybrook Research Institute (AUP #579) in Toronto, ON. The National Institutes of Health Guidelines for the Care and Use of Experimental Animals were met.

Tg(Sox9‐cre/ERT2)1Msan/J mouse (hemizygous, +/−) was purchased from the Jackson Laboratory (Bar Harbor, ME, USA, Stock No. 018829). The mouse was bred to B6.129X1‐Gt(ROSA)26Sortm1(EYFP)Cos/J mouse (homozygous, +/+, Bar Harbor, ME, USA, Stock No. 006148) to generate Sox9‐cre/ERT2^+/−^:ROSA26 EYFP^+/−^ offspring (F1). F1 mice were cross‐bred and F2 of Sox9‐cre/ERT2^+/−^: ROSA26 EYFP^+/+^ were selected for continuous breeding. Genotyping was performed following the protocol on the official website of the Jackson Laboratory, and the primers are listed in Table S1.

Eight‐ to eleven‐week‐old male mice with the genetic background of Sox9‐cre/ERT2^+/−^: ROSA26 EYFP^+/+^ were included for the animal experiments. Tamoxifen (Sigma) was dissolved at 20 mg/mL in corn oil (Sigma) and administered subcutaneously at a dosage of 100 mg/kg body weight. Tamoxifen was administered once daily for 3 consecutive days. Wild‐type mice of the same age and non‐tamoxifen control were also kept for baseline determination. The mice were randomly divided into sham and burned groups and received 30% total body surface area (TBSA) scald burn^18^ or sham treatment immediately after the first injection of Tamoxifen. The mice were killed on post‐burn day 2, 7, 14, 21, 28 and 42 (referred to as different observational groups). N = 6 for each group including sham control. Mice killed on post‐burn day 2 received 2 doses of tamoxifen injection.

### 2.2 Liver tissue collection and digestion

Upon killing, the inferior vena cava was cut and the whole liver was collected after brief portal vein perfusion with PBS (2 mL). The liver was weighed, and 2 small pieces of liver were taken and frozen immediately on dry ice and then stored at −80°C for gene expression and Western blot analyses. Another piece of liver tissue was fixed in 10% buffered formalin at 4°C overnight, transferred to 70% ethanol and then paraffin embedded for histology. The rest of the liver tissue was chopped into fine particles less than 1 mm^3^ and transferred to 5 mL digestion cocktail (200 U dispase (Sigma, Cat#4693), 270 mg Type I collagenase (Life Technologies, Cat#07912) in 100 mL DMEM with 1% Ab/Am) for cell staining and flow cytometry analysis and cell sorting.

### 2.3 Reagents and antibodies

Antibodies against CHOP (L63F7, mAb#2895), phospho‐eIF2α ((Ser51) D9G8, mAb#3398), eIF2α (#9722), ATF4 (D4B8, mAb#11815), BiP (#3183), HSP90 (E289, #4875), PARP (46D11, mAb#9532), IL‐1β (D3U3E, mAb#12703), phospho‐p38 MAPK (D3F9, mAb#4511), p38 MAPK (D13E1, mAb#8690), GAPDH (14C10, mAb#2118) and EpCAM (VU1D9, mAb#5447, Alexa Fluor^®^ 647 Conjugate) were purchased from Cell Signaling. Anti‐phospho‐IRE1α (Ser724, PA1‐16927) antibody was purchased from Thermo Scientific Inc Anti‐CPT1A (8F6AE9, ab128568), anti‐LXRα ([PPZ0412]‐ChIP Grade (ab41902)) and anti‐GFP (Ab5450) antibodie**s** were purchased from Abcam. Clarity Western ECL substrate was purchased from Bio‐Rad.

### 2.4 Western blotting

Liver homogenate lysates (50 μg of protein per well) were separated by 10% SDS‐PAGE gel, proteins were transferred to nitrocellulose membrane as previously described,^19^ and then, blots were probed using the antibodies listed above. Band intensities were detected, normalized and quantified with the Chemidoc and Image Lab 5.0 software (Bio‐Rad Laboratories). GAPDH was used as a loading control.

### 2.5 Cell staining and flow cytometry

Cells were incubated in digestion cocktail in 37°C for 40 minutes, then added equal volume of DMEM with 10% FBS and filtered through 40 μm strainer to a new tube. The cells were then washed with FCM buffer (0.5% BSA in HBSS). Cell count was performed with trypan blue using TC20TM automatic cell counter (Bio‐Rad Laboratories). 5 million cells (viability is between 30%‐50%) were transferred to flow tube and washed with FCM buffer. The cells were then blocked with CD16/32 on ice for 10 minutes followed by incubation with antibodies against EpCAM in FCM buffer on ice for 30 minutes. The sample was then washed once with FCM buffer and then top up with FCM buffer with DAPI. For flow cytometry analysis, all samples were run on the BD LSR II Flow Cytometer (BD Biosciences). 100 000 events were collected for each sample, and data were analysed using FlowJo (v. 8.7) software. Cell sorting was run on the BD FACS Aria IIu (BD Biosciences).

### 2.6 Immunofluorescent multi‐channel staining of liver

Antibody staining was performed as described previously.^19^, ^20^ Primary antibodies were the same as in Western blotting. The percentage of marker‐positive cells was determined by taking representative images and directly counting cell number by blindfolded third party. Cell enumerations for each experiment are listed in the text or figure legends.

### 2.7 Microarray transcriptomic analysis

Sorted cells were centrifuged, the pellet was dissolved in TRIzol (Thermo Fisher Scientific, Cat#15596026), total RNA was extracted using a QIAGEN kit (Cat#74104) according to the manufacturer's instructions, and expression profiles were compared using microarray analysis. For gene profile analysis, RNA quality was assessed with a Bioanalyzer (Agilent Technologies), and samples with an RNA integrity number (RIN) greater than 8.0 were included for array. cDNA was generated using QIAGEN kit (Cat#330451) and Affymetrix WT‐Pico Kit (Cat#74134) and hybridized onto the Affymetrix Mouse Gene 2.0 ST chips (Cat#901169). Analysis of gene expression was performed using Parktec Genotyping Suite for gene level differential expression analysis and Ingenuity Systems Software for canonical signalling pathway analysis (QIAGEN Bioinformatics). Filter criteria for positive signals are folder changes greater than 2 and ANOVA *P*‐value < .05.

### 2.8 Statistical analysis

The statistical analysis was performed using Prism version 5.01 (GraphPad Software). One‐way ANOVA with Bonferroni's multiple comparison test was used unless otherwise specified, and *P* < .05 was considered statistically significant.

## 3 RESULTS

### 3.1 Increased proliferation of PDPCs contributes to hepatomegaly after major burn injury

**Figure 1:**
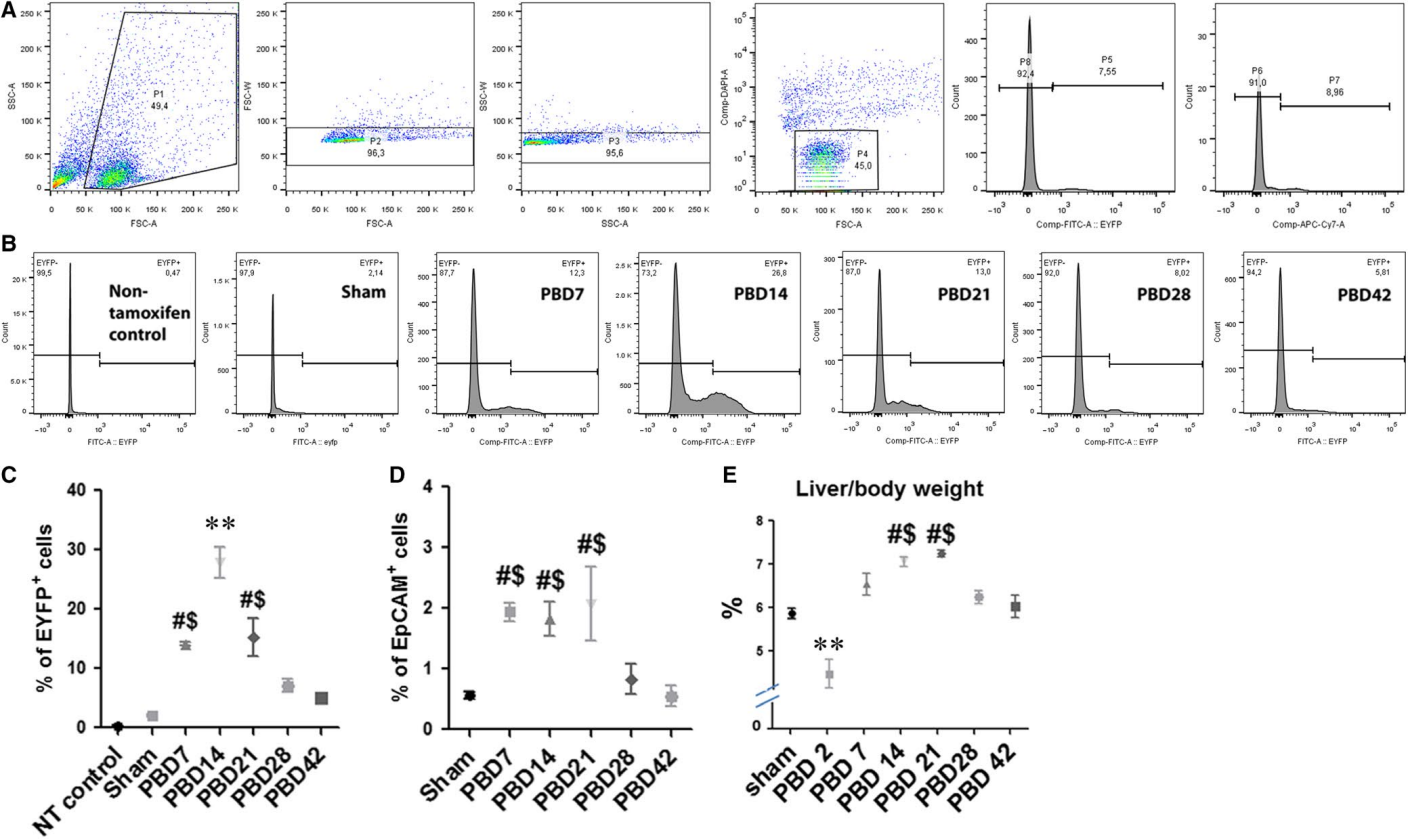
Flow cytometry analysis indicates increased proliferation of the PDPCs contributing to hepatomegaly after thermal injury. (A) Gating of hepatocytes of wild‐type mice, non‐tamoxifen–treated control and hepatocytes isolated from Sox9‐cre/ERT2^+/−^:ROSA26 EYFP^+/+^ mice 7 days post‐burn were presented. 3 groups of cells were separated: EYFP^‐^ (P8), EpCAM^‐^/EYFP^+^ (P6) and EpCAM^+^/EYFP^+^ (P7). P5 represented EYFP^+^ cells which are PDPCs in total including progenitor cells (P7) and progenitor cell derived hepatocytes (P6). (B) Representative spectrum images of EYFP^+^ cells versus total hepatocytes in mice after burn injury. (C,D) Statistical analysis of P5 (C) and P7 (D) versus total hepatocytes in different groups. (E) The comparison of liver/body weight ratio among the groups. Data are presented as means ± SEM. **P < .01 vs all other groups, ^#^ *P* < .05 vs sham, ^$^ *P* < .05 vs PBD42 group. N = 6 animals per group including non‐tamoxifen control, sham and different time‐point post‐burn

To specifically lineage‐trace the proliferation of the PDPCs in the liver after thermal injury, we generated Sox9‐cre/ERT2:ROSA26 EYFP mice in which the expression of EYFP in PDPCs is inducible upon tamoxifen treatment upon expression of Sox9. We optimized the protocol for the tamoxifen treatment by comparing different dosage of tamoxifen treatment with either intraperitoneal or subcutaneous injection. Flow cytometry analysis demonstrated that subcutaneous injection of tamoxifen for 3 consecutive days induced most consistent expression of EYFP (Figure S1A) which is supported by immunofluorescent staining of the liver tissue sections against anti‐GFP antibody (Figure S1B). By initiating the tamoxifen injection at the time when the mice were subjected to 30% total body surface area (TBSA) scald burn, we were able to lineage‐trace the fate of PDPCs after the thermal injury by checking the EYFP^+^ cells. When harvesting the cells, we performed cell staining of Alexa Fluor^®^ 647 Conjugated anti‐EpCAM (VU1D9) mouse monoclonal antibody to distinguish between PDPCs and PDPC‐derived hepatocytes^21^, ^22^ for the flow cytometry study (Figure 1A). We observed significantly increased cell population of the PDPCs and PDPC‐derived hepatocytes (total EYFP^+^ cells) in post‐burn day (PBD) 7, 14 and 21 as compared with sham (Figure 1B,C), peaking at around PBD14. Together with the significant increase in the EYFP^+^/EpCAM^+^ cells in PBD7, 14 and 21 (Figure 1D), we observed the increased proliferation and differentiation of PDPCs after thermal injury.

**Figure 2:**
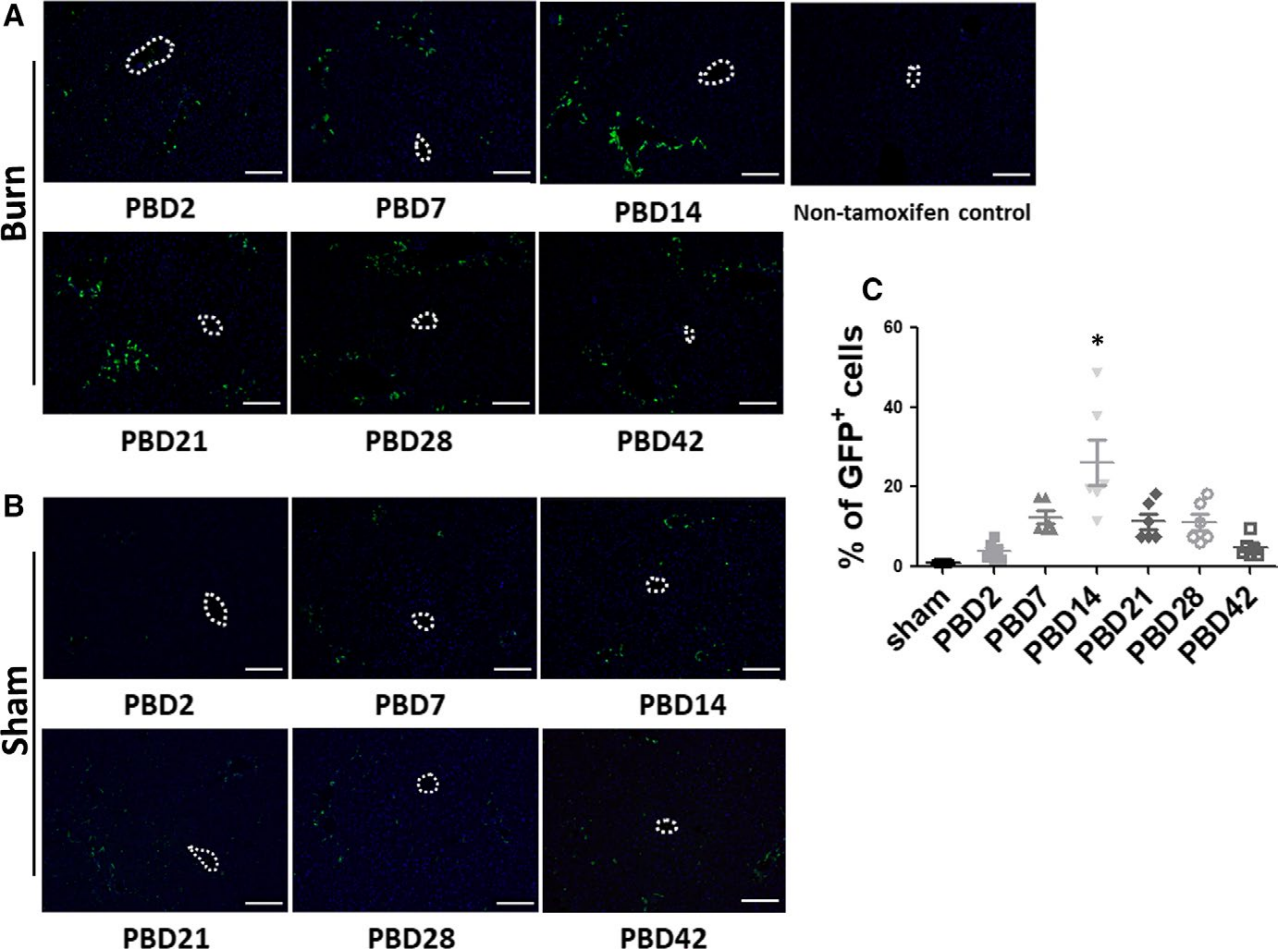
Histological time course study demonstrates proliferation of PDPCs after thermal injury. (A,B) Representative images of immunofluorescent staining of GFP in the liver sections of mice at different time‐points after thermal injury (A) as compared with the sham animals (B). (C) Statistical analysis of the positive cell counts in (A,B). *P < .01 vs all other groups. Dotted circles depict central veins. N = 6 in each group of burn and sham in different time‐points. Scale bar = 100 μm

To test whether such proliferation of PDPCs contributes to the increase in the hepatic parenchyma, we measured the weight of the whole liver of the mice and compared with the body weight of the mice when killed. As the body weight of the mice was generally stable during the whole observation period (Figure S2), the concomitant significant increase in liver/body weight ratio around PBD14 and 21 indicated hepatomegaly in this period of time (Figure 1E). We also performed immunofluorescent staining of the liver tissue sections with anti‐GFP antibody to examine the distribution of the EYFP^+^ cells in the liver tissue (Figure 2A,B). It was clearly demonstrated the increase in the EYFP^+^ cells around the portal triads after thermal injury. The statistical analysis of the positive cell counts showed consistent results with the flow cytometry analysis (Figure 2C).

Interestingly, while the proliferation of PDPCs peaked around PBD14 and significantly attenuated afterwards (Figure 1C), the increase in the liver mass peaked around PBD21 (Figure 1E). Also, we were unable to see the pattern of streaming of liver regeneration from portal triads to central venule systems (Figure 2A). These results suggest that the liver regeneration after major burn injury is not from a single type of progenitor cells but via an orchestrated proliferation of both PDPCs and mature hepatocytes.

### 3.2 The hepatic cellular stress response is implicated in signalling the proliferation of PDPCs after major burn injury

**Figure 3:**
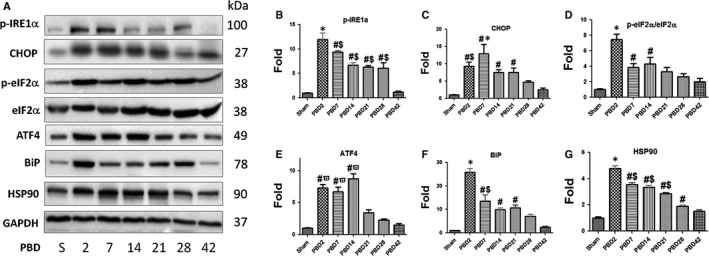
Hepatic cellular stress response after thermal injury. (A) Representative images of Western blot of multiple cellular stress markers in whole liver lysate in different time after burn injury as compared with sham. (B) to (G) Densitometric analysis of the Western blots of phospho‐IRE1α (B), CHOP (C), phospho‐eIF2α/eIF2α (D), ATF4 (E), BiP (F) and HSP90 (G). Data are presented as means ± SEM. *P < .05 as compared with all the other groups. ^#^ *P* < .05 as compared with Sham. ^$^ *P* < .05 as compared with PBD42. ^ϖ^ *p* < 0.05 as compared with PBD21, 28 and 42. N = 6 animals per group including non‐tamoxifen control, sham and different time‐point post‐burn

**Figure 4:**
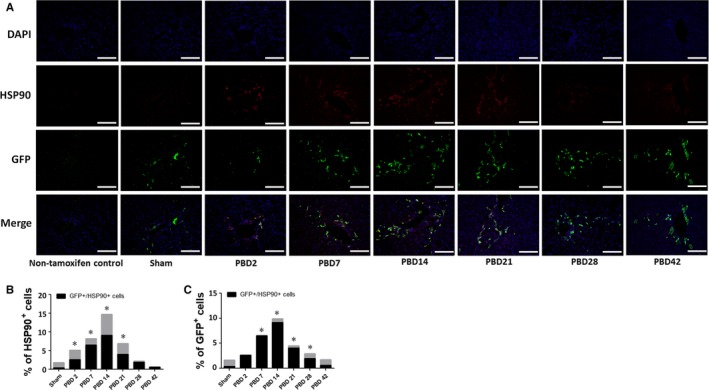
Histological study demonstrates the correlation of hepatic cellular stress response with the increased proliferation of the PDPCs after thermal injury. (A) Representative images of immune‐fluorescent double staining of HSP90 and GFP of liver tissue sections of the mice at different time‐points after burn injury as compared with sham. (B,C) Statistical analysis of the positive cell counts of HSP90 + cells (B) and GFP + cells (C) in the liver tissue sections after burn injury as compared with sham. N = 6 animals per group including non‐tamoxifen control, sham and different time‐point post‐burn. Scale bar = 50 μm. *P < .05 as compared with sham

In our previous studies, we have demonstrated the augmented hepatic ER stress and subsequent increase of apoptosis in the liver in the early post‐burn period in several rodent thermal injury models.^23^, ^24^ In the current study, we examined the expression of multiple cellular stress markers including phospho‐IRE1α, CHOP, phospho‐eIF2α versus eIF2α, ATF4, BiP and HSP90 in the liver tissue by Western blotting (Figure 3A) of whole liver lysate samples and densitometry analysis showed that significant hepatic stress response occurred and culminated between PBD2 to PBD7, persisted to PBD21 and resolved after PBD28 to almost normal at PBD42, the end‐point of our observation (Figure 3B‐G). Opposing to the general understanding that ER stress induces apoptosis especially within the context of overall increased levels of ATF4, CHOP and eIF2α phosphorylation, the chronological consistency between the hepatic stress response and PDPCs proliferation implicated their correlation after burn injury. In order to verify the types of cells mainly carrying the stress response signals, we performed immunofluorescent double staining of the liver sections against anti‐HSP90 and anti‐GFP antibodies and we found significant co‐localization of the HSP90^+^ and GFP^+^ cells in the liver after burn injury (Figure 4A). Chronologically, we observed (a) a small part of HSP90^+^ cells were GFP^+^ on PBD2; (b) an increased portion of HSP90^+^ cells were GFP^+^ and almost all GFP^+^ cells are HSP90^+^ on PBD7; (c) the total number of GFP^+^/HSP90^+^ cells peaked at PBD14; and (d) as the cellular stress response resolved after PBD21 which was indicated by the decrease in the number of HSP90^+^ cells, the number of GFP^+^ cells was still higher than sham animals group through PBD28 (Figure 4B,C), depicting the correlation between hepatic cellular stress and PDPCs proliferation.

### 3.3 Transcriptomic analysis revealed enhanced pro‐inflammatory signalling and hypermetabolism in PDPC and their progeny after burn injury

**Figure 5:**
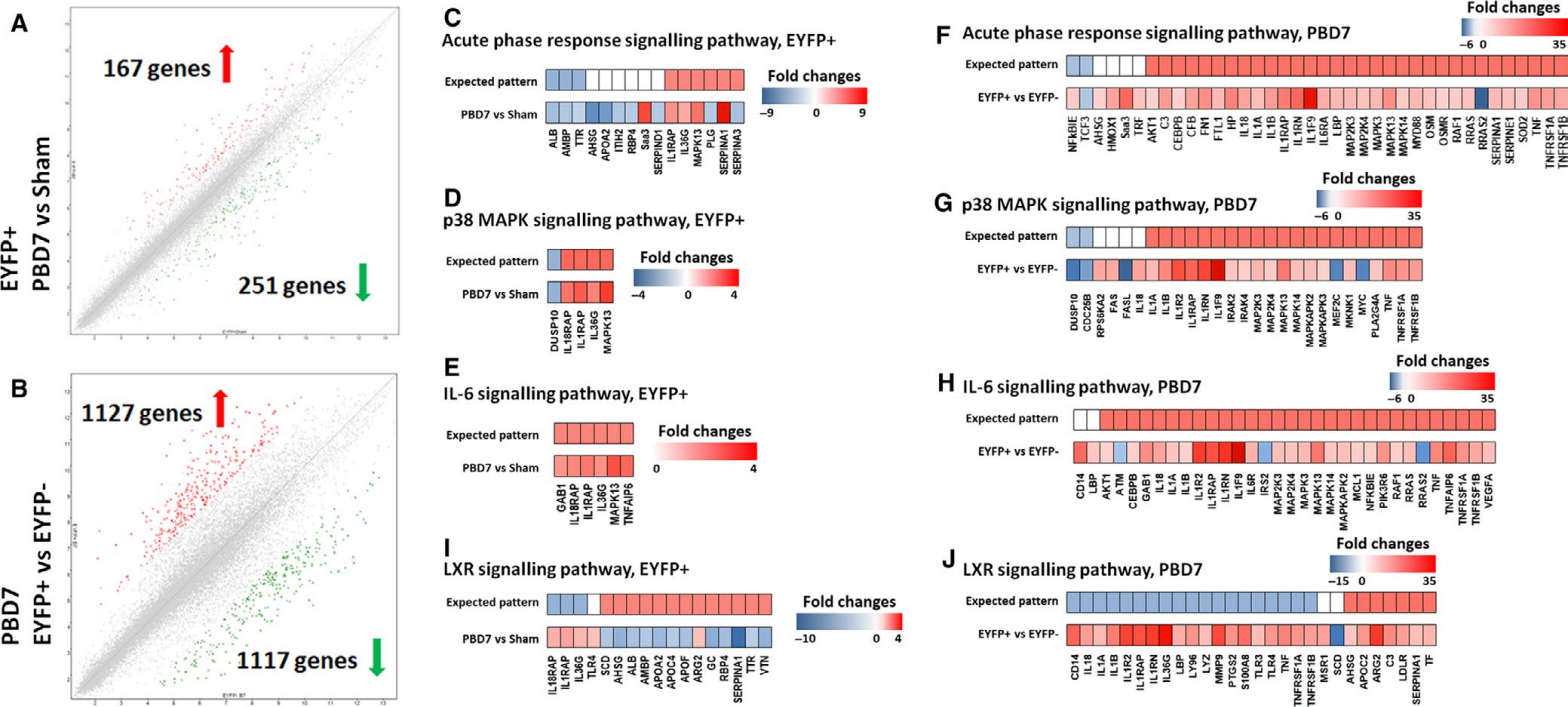
Transcriptomic analysis demonstrates up‐regulation of the acute phase response, p38 MAPK and IL‐6 signalling pathways and down‐regulation of the LXR/RXR signalling pathway in PDPCs after thermal injury. (A,B) By setting up the filter criteria as fold changes (linear) <−2 (green arrows) or >2 (red arrows); and ANOVA *p*‐value (condition pair) < .05, transcriptomic analysis indicates significant changes in the levels of gene expression of EYFP^+^ cells on PBD7 versus sham (A) and significant changes in the level of gene expression of EYFP^+^ cells versus EYFP^‐^ cells on PBD7 (B). (C,D,E) Canonical signalling pathway analysis demonstrates up‐regulated acute phase response (C), p38 MAPK (D) and IL‐6 (E) signalling pathways of EYFP^+^ cells on PBD7 versus sham. (F,G,H) Up‐regulation of these signalling pathways are also seen in EYFP^+^ cells when compared with EYFP^‐^ cells of the same mice on PBD7. (I,J) Concomitant down‐regulation of LXR signalling pathway is seen in EYFP^+^ cells of PBD7 group when compared with both EYFP^+^ cells of sham (I) and EYFP^‐^ cells of the same mice (J). From panel (C) to (J), the heat maps indicate the fold changes in the expression of the key genes of each signalling pathway. The upper bar of each panel indicates the expected pattern of the gene expression when the signalling pathway is activated and the lower bar is the fold changes of the gene expression in EYFP^+^ cells of PBD7 mice versus sham (C,D,E,I) and EYFP^+^ versus EYFP^‐^ cells in PBD7 mice (F,G,H,J). N = 3 per group

We next asked whether and how the increased number of the PDPCs contributes to the persistent pro‐inflammatory response and hypermetabolism after burn injury. We first performed microarray analysis to compare the transcriptome of (a) PDPCs and PDPC‐derived hepatocytes before and 7 days after burn injury; and (b) PDPCs and PDPC‐derived hepatocytes versus mature hepatocytes on PBD7. We found that, among the 34 472 genes analysed, 418 genes are differentially expressed between the EYFP^+^ cells of sham and mice of PBD7 group (Figure 5A); 2344 genes are differentially expressed between the EYFP^+^ (PDPCs and their progeny) and EYFP^‐^ (mature hepatocytes) cells from the same liver of the mice of PBD7 group (Figure 5B).

We then conducted signalling pathway analysis to unravel how these differentially expressed genes implicate the changes in cell physiology. There are 5 up‐regulated signalling pathways and 11 down‐regulated signalling pathways in the EYFP^+^ cells on PBD7 as compared with that of sham (Table S2). When compared the EYFP^+^ cells with the EYFP^‐^ cells on PBD7, we found 52 up‐regulated signalling pathways and 12 down‐regulated signalling pathways (Table S3). Among these, acute phase response signalling, IL‐6 signalling and p38 MAPK signalling are pathways of note as the activation of these pathways in PDPCs and PDPC‐derived hepatocytes after burn injury was not only significantly demonstrated as compared with that of sham (Figure 5C,D,E) but also more robust as compared with mature hepatocytes under the same condition (Figure 5F,G,H).

### 3.4 Down‐regulation of the LXR/RXR signalling pathway in PDPCs early post‐burn

Concomitantly, we noticed significant down‐regulation of the LXR/RXR signalling pathway in the PDPCs and PDPC‐derived hepatocytes post‐burn as compared with sham and with the mature hepatocytes at the same time‐point post‐burn (Figure 5I,J), implicating the impairment of lipid homeostasis in the PDPCs and PDPC‐derived hepatocytes and its contribution to the overwhelming pro‐inflammatory response in the liver as a result of the increased proliferation and differentiation of the PDPCs.^25^

**Figure 6:**
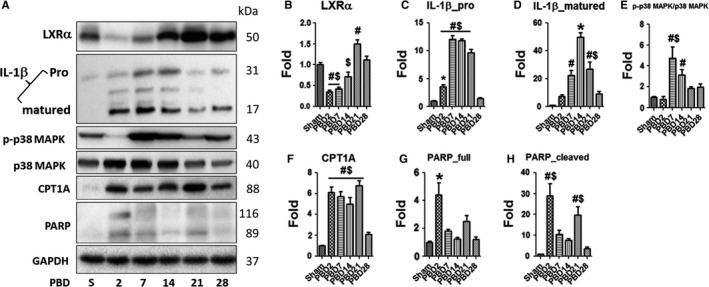
Up‐regulated hepatic acute phase response and p38 MAPK signalling followed the decrease in LXRα expression and correlated with increased lipid oxidation and cell damage and replenishment in the liver after thermal injury. (A) Representative images of the Western blots of LXRα, pro‐IL‐1β and matured IL‐1β, phospho‐ and total p38 MAPK, CPT1A and PARP in the whole liver lysate of mice post‐burn injury as compared with sham, with GAPDH as sample loading control. (B) to (H) Densitometric analysis of the expression of LXRα (B), pro‐IL‐1β (C) and matured IL‐1β (D), phospho‐p38 MAPK/p‐38 MAPK ratio (E), CPT1A (F) and full and cleaved form of PARP (G and H) in the liver tissue of mice after burn injury versus sham. Data are presented as means ± SEM. *P < .05 as compared with all the other groups. ^#^ *P* < .05 as compared with Sham. ^$^ *P* < .05 as compared with PBD28. N = 6 animals per group including non‐tamoxifen control, sham and different time‐point post‐burn

To confirm our above findings of the activation or inhibition of the signalling pathways and their correlation with the post‐burn pro‐inflammatory response and metabolic activation, we examined the levels of the expression of some key modulators or effectors in the liver tissue including LXRα, IL‐1β, phospho‐p38 MAPK, p38 MAPK, CPT1A and PARP (Figure 6A). The decreased levels of expression of LXRα, as well as the increased levels of expression of pro‐IL‐1β, matured IL‐1β and phospho‐p38 MAPK, were all consistent with the microarray transcriptomic data.

### 3.5 Persistent hepatic pro‐inflammatory response and hypermetabolism, as well as implicated increased turnover of liver parenchyma after major thermal injury

Specifically, the level of hepatic LXRα significantly decreased between PBD2 to PBD14 with the concomitant presence of the cellular stress response (Figure 3) and then significantly increased around PBD21 and PBD28 along with the resolving of the inflammation and the restoration of homeostasis after the major burn injury (Figure 6B). We observed rapid and significant increase in the expression of hepatic pro‐IL‐1β from PBD2 to PBD21, resolving to the level of sham animals on PBD28 (Figure 6C). There seemed to be delayed and more temporal increase in the level of hepatic matured IL‐1β which peaked around PBD14 (Figure 6D). Similarly, the level of the hepatic phospho‐p38 MAPK versus total p38 MAPK increased significantly around PBD7 to PBD14 (Figure 6E) which, together with the changes in the expression of hepatic LXRα and IL‐1β, implicated the activation of the immunological responses and inflammatory signalling between PBD2 to PBD14 or 21.

Moreover, we measured the level of expression of CPT1A, which is the rate‐limiting regulator of hepatic β‐oxidation,^26^ and it was demonstrated that there was increased β‐oxidation from PBD2 to PBD21, reflecting the increased energy demand and expenditure after burn injury (Figure 6F). We also examined the expression level of PARP in the liver tissue (Figure 6G,H). The significant increase in the level of PARP around PBD2 suggested liver cell damage and increased apoptosis in early post‐burn period. Interestingly, we observed a bi‐phasic increase in cleaved hepatic PARP post‐burn. The second phase of the increase in cleaved PARP was around PBD21, which is concomitant with the attenuation of the PDPCs proliferation from PBD14 to PBD21, implicating the replenishment of PDPC‐derived hepatocytes by the proliferating mature hepatocytes. Such concomitance of PDPCs’ turnover and the restoration of the immunological and metabolic homeostasis further suggested the contribution of PDPCs and their proliferation to the pro‐inflammatory response and hypermetabolism after major burn injury.

## 4 DISCUSSION

In the current study, using the reporter mice strain of Sox9‐cre/ERT2:ROSA26 EYFP, we were able to lineage‐trace the proliferation and differentiation of PDPCs after burn injury. By flow cytometry analysis, we observed increased proliferation of PDPCs which peaks around two weeks post‐burn. The pool of progenitors also expands temporarily from one to three weeks post‐burn which is concomitant with the increased proliferation and differentiation of PDPCs.

It has been well accepted that severe liver damage and the impairment of the renewal of hepatic parenchyma by self‐duplication of mature hepatocytes trigger the proliferation of the PDPCs for the liver regeneration.^21^ Here, we have demonstrated that 30%TBSA scald burn is an insult strong enough to activate PDPCs proliferation. It has also been suggested that ER stress and subsequent activation of unfolded protein response (UPR) enable the cell to either resolve stress or initiate apoptosis and while primary stem cells are more prone to cell death under stress, closely related progenitor cells exhibit more adaptive response leading to their survival.^27^ By further investigating the chronological feature of hepatic cellular stress response and PDPCs proliferation after burn injury, we demonstrated the correlation of cellular stress response and the activation of the proliferation of the PDPCs. Moreover, immunofluorescent double staining of liver tissue sections with antibodies against GFP (to label EYFP^+^ cells) and HSP90 (cellular stress marker) showed the increased number of GFP^+^/HSP90^+^ cells around portal venule from PBD2 to PBD28, confirming the resistance of cell death signalling and the activation of the proliferation of the PDPCs after burn injury. Nevertheless, how such stress signals trigger the proliferation and differentiation of the PDPCs still warrants further investigation.

Two phenomena implicate the orchestrated cell proliferation of both PDPCs and mature hepatocytes, contributing to the hepatomegaly after burn injury:

1. The proliferation of the PDPCs post‐burn peaks around PBD14 with the EYFP^+^ cell population over 20% of the total hepatocytes count. The drastic increase in the EYFP^+^ cells seen on PBD7 and PBD14 is concomitant with the increase in the liver/body weight ratio. However, the EYFP^+^ cell population decreased from over 20% to around 15% of the total hepatic parenchyma from PBD14 to PBD21 and the liver/body weight ratio peaks around PBD21 within the context of stable or slightly increased body weight. This indicates that cells other than PDPCs also contribute to the increased liver mass from PBD14 to PBD21.
2. When examining the histological pattern of the liver regeneration after burn injury, we did not see the pattern of streaming of the hepatocytes from portal triads to central venule systems. We found most of the EYFP^+^ cells are along the portal venule from PBD2 to PBD7, disseminating to the liver plates around PBD14 and PBD21, but seldom stretching out to the central venule system afterwards.

To better explain the above phenomena, we speculate that the liver regeneration in the early post‐burn period is mainly via the proliferation of PDPCs which is activated by significant cellular stress response and liver damage, whereas two to three weeks after injury, with the approaching of the wound closure and the restoration of total body homeostasis, the cellular stress response is attenuating and the liver regeneration is gradually taken over by the physiological self‐renewal of the mature hepatocytes.^28^

Nevertheless, we noticed that not only cell proliferation but also fat infiltration might contribute to the increase of liver mass in early post‐burn period while such a fat infiltration subsided within two weeks post‐injury (Figure S3). Moreover, when we determined the hepatic PARP level after burn injury, we noticed a bi‐phasic increase of the expression of cleaved form of PARP around both PBD2 and PBD21, indicating increased apoptosis at these two time‐points post‐burn. It is clear that the first phase of the increase correlates with acute hepatic stress response and increased apoptosis after burn injury which is consistent with the increased expression of the multiple cellular stress markers.^24^, ^29^ The second phase of the increase in the cleaved PARP is concomitant with the decrease in the EYFP^+^ cells from around 25% on PBD14 to 15% on PBD21 and 10% on PBD28, implicating the clearance of the PDPCs and PDPC‐derived hepatocytes when the homeostasis is finally restored after the injury.

Transcriptomic analysis in the current study reveals the significant activation of pro‐inflammatory and stress signalling pathways, including acute phase response signalling pathway, IL‐6 signalling pathway, p38 MAPK signalling pathway, in PDPCs and their progeny after burn injury as compared with either the sham control or the mature hepatocytes at the same time‐point after burn injury. We demonstrated the consequence of the activation of such pro‐inflammatory and stress signalling pathways post‐burn at the organ level by examining the expression of the key regulators or effectors of the pathways in the liver, including the pro‐IL1β and its mature form, as well as total and phospho‐p38 MAPK.

Recently, there is growing recognition of immune mediators, TNFα, IL‐1β and IL‐6, acting as metabolic hormones 30 enhancing metabolic activities by increasing the energy expenditure and substrate consumption.^4^ It is well accepted that the p38 MAPK signalling pathway can be activated by a wide range of cellular stress signals and is critical for immune and immunological responses.^31^ Also, the synergistic interaction among these signalling pathways is evident.^32^ Taking together, from the point of view of translational medicine, it is appropriate to consider the expanded population of PDPCs and the activation of the above signalling pathways in these cells as the potential contributing factors to the prolonged inflammatory response and hypermetabolism seen in major burned patients.

Based on our observation, the duration of the pro‐inflammatory response and metabolic derangement in the burned mice is around 3 to 4 weeks, peaking at around 2 weeks post‐burn. As the maturation rate of the mice aged 1 to 6 month is about 45 times that of human,^33^ three weeks in mice could be roughly equivalent to 2 years in humans. The duration of the pro‐inflammatory response and metabolic derangement we observed in this burned mice study is thus consistent with the clinical observations of the persistent pro‐inflammatory states and hypermetabolism in major burned patients.

We found the down‐regulation of the hepatic LXR/RXR signalling pathway concomitant with the activation of the above pro‐inflammatory pathways after burn injury by both the transcriptomic analysis and determination of the changes in the level of expression of LXRα in the liver tissue. It is interesting to notice that, on the one hand, LXR signalling is inhibitory to inflammatory responses^34^ and thus the down‐regulation of the LXR signalling pathway at least correlates with, if not contributes to the activation of the pro‐inflammatory responses;^35^ on the other hand, LXR signalling is pivotal to lipid homeostasis in mammals and the repression of the LXR signalling implicates impaired lipid metabolism post‐burn.^36^

More importantly, this may suggest a novel therapeutic target for the care of the severe trauma patients early after the injury. It will be interesting to see whether early and short‐term application of LXR agonists to those major trauma patients can be beneficial to the control of overwhelming stress response and pro‐inflammatory response, as well as the amelioration of the subsequent and persistent metabolic derangement. There have been several LXR agonists in different phases of clinical trials for the treatment of atherosclerosis. However, a major issue of concern is their undesirable effects on hepatic lipogenesis and thus the increased risk of hepatic steatosis if they are used for long time.^25^ We would propose clinical trial to see whether short‐term administration of these LXR agonists to major burn patients in their early post‐injury phase would be beneficial, safe and feasible.

In this regard, additional consolidated animal experiments should be conducted beforehand including examining the response of LXR knockout mice on burn injury and LXR agonist administration to the Sox9‐cre/ERT2: EYFP+ mice after burn injury. Especially, when the time‐points of the observation of the animal experiments are chosen, it is very challenging to accurately define the equivalency of what we see in the animals to the human pathology. Multiple time‐points should thus be included in the future studies in order to unravel the fate of Sox9‐positive cells at the different time‐points post‐thermal injury. Specifically, inactivation of LXR gene in the Sox9‐positive cells shed light on the role of this molecule in the post‐traumatic hepatic responses. Moreover, as microarray analysis can only determine the fixed number of pre‐defined genes (34 472 genes in the current study), the read‐out of the analysis is not objective and inclusive. If RNA sequencing can be done, more information could be available including long non‐coding RNAs, micro‐RNAs and RNA modifications such as splicing and cleavage. These are all very important for the mechanistic study of the gene transcriptional regulation and control. Also, more accurate information could be available as the copy numbers can be collected directly without the possible skew of the information via PCR amplification. Also, we can look forward to more comprehensive understanding of the dynamic changes of the signalling pathways by transcriptomic profiling if PBD14, 21, 28 and 42 samples can be included for the analysis.

In conclusion, hepatic cellular stress responses and cell damage stimulates proliferation and differentiation of PDPCs with activated pro‐inflammatory and stress signalling, contributing to the persistent pro‐inflammatory response and metabolic activation after major burn injury. LXRα agonists may have potential therapeutic effects to ameliorate such pro‐inflammatory response and hypermetabolism if administered early after the injury.

## CONFLICT OF INTEREST

The authors confirm that there are no conflicts of interest.

## AUTHOR CONTRIBUTIONS

LD, SA and MGJ contributed to conceptualization; LD and SA contributed to methodology; LD and YY contributed to investigation; LD contributed to writing–original draft; SA and MGJ contributed to resources and writing–review and editing; MGJ contributed to funding acquisition and supervision.

## Data Availability

The data that support the findings of this study are available from the corresponding author upon reasonable request.

## References

[1] Jeschke MG , Boehning D . Endoplasmic reticulum stress and insulin resistance post‐trauma: similarities to type 2 diabetes. J Cell Mol Med. 2012;16:437‐444. 21812914 10.1111/j.1582-4934.2011.01405.x PMC3217064

[2] Jeschke MG , Finnerty CC , Herndon DN , et al. Severe injury is associated with insulin resistance, endoplasmic reticulum stress response, and unfolded protein response. Ann Surg. 2012;255:370‐378. 22241293 10.1097/SLA.0b013e31823e76e7 PMC3395076

[3] Long CL , Schaffel N , Geiger JW , Schiller WR , Blakemore WS . Metabolic response to injury and illness: estimation of energy and protein needs from indirect calorimetry and nitrogen balance. JPEN. 1979;3:452‐456. 10.1177/014860717900300609 575168

[4] Porter C , Tompkins RG , Finnerty CC , et al. The metabolic stress response to burn trauma: current understanding and therapies. Lancet. 2016;388:1417‐1426. 27707498 10.1016/S0140-6736(16)31469-6 PMC5753602

[5] Jeschke MG , Barrow RE , Herndon DN . Extended hypermetabolic response of the liver in severely burned pediatric patients. Arch Surg. 2004;139:641‐647. 15197091 10.1001/archsurg.139.6.641

[6] Jeschke MG , Gauglitz GG , Kulp GA , et al. Long‐term persistance of the pathophysiologic response to severe burn injury. PLoS ONE. 2011;6:e21245. 21789167 10.1371/journal.pone.0021245 PMC3138751

[7] Jeschke MG . The hepatic response to thermal injury: is the liver important for postburn outcomes? Mol Med. 2009;15:337‐351. 19603107 10.2119/molmed.2009.00005 PMC2710295

[8] Huch M , Dolle L . The plastic cellular states of liver cells: are EpCAM and Lgr5 fit for purpose? Hepatology. 2016;64:652‐662. 26799921 10.1002/hep.28469 PMC4973669

[9] Michalopoulos GK . Liver regeneration. J Cell Physiol. 2007;213:286‐300. 17559071 10.1002/jcp.21172 PMC2701258

[10] Sadri AR , Jeschke MG , Amini‐Nik S . Advances in liver regeneration: revisiting hepatic stem/progenitor cells and their origin. Stem Cell Intern. 2016;2016:7920897. 10.1155/2016/7920897 PMC4699025 26798363

[11] Tarlow BD , Pelz C , Naugler WE , et al. Bipotential adult liver progenitors are derived from chronically injured mature hepatocytes. Cell Stem Cell. 2014;15:605‐618. 25312494 10.1016/j.stem.2014.09.008 PMC4254170

[12] Yanger K , Knigin D , Zong Y , et al. Adult hepatocytes are generated by self‐duplication rather than stem cell differentiation. Cell Stem Cell. 2014;15:340‐349. 25130492 10.1016/j.stem.2014.06.003 PMC4505916

[13] Yovchev MI , Grozdanov PN , Zhou H , et al. Identification of adult hepatic progenitor cells capable of repopulating injured rat liver. Hepatology. 2008;47:636‐647. 18023068 10.1002/hep.22047

[14] Font‐Burgada J , Shalapour S , Ramaswamy S , et al. Hybrid periportal hepatocytes regenerate the injured liver without giving rise to cancer. Cell. 2015;162:766‐779. 26276631 10.1016/j.cell.2015.07.026 PMC4545590

[15] Hoehme S , Brulport M , Bauer A , et al. Prediction and validation of cell alignment along microvessels as order principle to restore tissue architecture in liver regeneration. Proc Natl Acad Sci USA. 2010;107:10371‐10376. 20484673 10.1073/pnas.0909374107 PMC2890786

[16] Turner R , Lozoya O , Wang Y , et al. Human hepatic stem cell and maturational liver lineage biology. Hepatology. 2011;53:1035‐1045. 21374667 10.1002/hep.24157 PMC3066046

[17] Cardinale V , Wang Y , Carpino G , et al. The biliary tree–a reservoir of multipotent stem cells. Nat Rev Gastro Hepatol. 2012;9:231‐240. 10.1038/nrgastro.2012.23 22371217

[18] Auger C , Sivayoganathan T , Abdullahi A , Parousis A , Jeschke MG . Hepatic mitochondrial bioenergetics in aged C57BL/6 mice exhibit delayed recovery from severe burn injury. Biochim Biophys Acta. 2017;1863:2705‐2714. 10.1016/j.bbadis.2017.07.006 PMC5659908 28711594

[19] Diao L , Patsouris D , Sadri AR , Dai X , Amini‐Nik S , Jeschke MG . Alternative mechanism for white adipose tissue lipolysis after thermal injury. Mol Med. 2015;21:959‐968. 26736177 10.2119/molmed.2015.00123 PMC4818253

[20] Amini‐Nik S , Cambridge E , Yu W , et al. beta‐Catenin‐regulated myeloid cell adhesion and migration determine wound healing. J Clin Invest. 2014;124:2599‐2610. 24837430 10.1172/JCI62059 PMC4089463

[21] Miyajima A , Tanaka M , Itoh T . Stem/progenitor cells in liver development, homeostasis, regeneration, and reprogramming. Cell Stem Cell. 2014;14:561‐574. 24792114 10.1016/j.stem.2014.04.010

[22] Dolle L , Theise ND , Schmelzer E , et al. EpCAM and the biology of hepatic stem/progenitor cells. Am J Physiol Gastro Liv Physiol. 2015;308:G233‐G250. 10.1152/ajpgi.00069.2014 PMC4329473 25477371

[23] Diao L , Auger C , Konoeda H , Sadri AR , Amini‐Nik S , Jeschke MG . Hepatic steatosis associated with decreased beta‐oxidation and mitochondrial function contributes to cell damage in obese mice after thermal injury. Cell Death Dis. 2018;9:530. 29748608 10.1038/s41419-018-0531-z PMC5945855

[24] Jeschke MG , Gauglitz GG , Song J , et al. Calcium and ER stress mediate hepatic apoptosis after burn injury. J Cell Mol Med. 2009;13:1857‐1865. 20141609 10.1111/j.1582-4934.2008.00644.x PMC2855735

[25] Hong C , Tontonoz P . Liver X receptors in lipid metabolism: opportunities for drug discovery. Nat Rev Drug Disc. 2014;13:433‐444. 10.1038/nrd4280 24833295

[26] Lee K , Kerner J , Hoppel CL . Mitochondrial carnitine palmitoyltransferase 1a (CPT1a) is part of an outer membrane fatty acid transfer complex. J Biol Chem. 2011;286:25655‐25662. 21622568 10.1074/jbc.M111.228692 PMC3138250

[27] van Galen P , Kreso A , Mbong N , et al. The unfolded protein response governs integrity of the haematopoietic stem‐cell pool during stress. Nature. 2014;510:268‐272. 24776803 10.1038/nature13228

[28] Tanimizu N , Mitaka T . Re‐evaluation of liver stem/progenitor cells. Organogenesis. 2014;10:208‐215. 24451175 10.4161/org.27591 PMC4154955

[29] Marshall AH , Brooks NC , Hiyama Y , et al. Hepatic apoptosis postburn is mediated by c‐Jun N‐terminal kinase 2. Shock. 2013;39:183‐188. 23324888 10.1097/SHK.0b013e31827f40ab PMC3552323

[30] Hotamisligil GS . Inflammation, metaflammation and immunometabolic disorders. Nature. 2017;542:177‐185. 28179656 10.1038/nature21363

[31] Cuenda A , Rousseau S . p38 MAP‐kinases pathway regulation, function and role in human diseases. Biochim Biophys Acta. 2007;1773:1358‐1375. 17481747 10.1016/j.bbamcr.2007.03.010

[32] Yang HT , Cohen P , Rousseau S . IL‐1beta‐stimulated activation of ERK1/2 and p38alpha MAPK mediates the transcriptional up‐regulation of IL‐6, IL‐8 and GRO‐alpha in HeLa cells. Cell Signal. 2008;20:375‐380. 18065201 10.1016/j.cellsig.2007.10.025

[33] Flurkey K , Currer JM , Harrison DE , et al. The mouse in aging research In: Fox JG, ed. The Mouse in Biomedical Research, 2nd ed Burlington, MA: American College Laboratory Animal Medicine (Elsevier); 2007:637‐672.

[34] Schulman IG . Liver X receptors link lipid metabolism and inflammation. FEBS Lett. 2017;591(19):2978‐2991. 28555747 10.1002/1873-3468.12702 PMC5638683

[35] Gabbi C , Warner M , Gustafsson JK . Action mechanisms of liver X receptors. Biochem Biophys Res Commun. 2014;446(3):647‐650. 24300092 10.1016/j.bbrc.2013.11.077

[36] Kidani Y , Bensinger SJ . Liver X receptor and peroxisome proliferator‐activated receptor as integrators of lipid homeostasis and immunity. Immunol Rev. 2012;249:72‐83. 22889216 10.1111/j.1600-065X.2012.01153.x PMC4007066

