# Supplementary figures and images for: Increased proliferation of hepatic periportal ductal progenitor cells contributes to persistent hypermetabolism after trauma
Source: J Cell Mol Med. 2019 Dec 3;24(2):1578–87. doi: 10.1111/jcmm.14845 (PMC6991656; doi:10.1111/jcmm.14845)

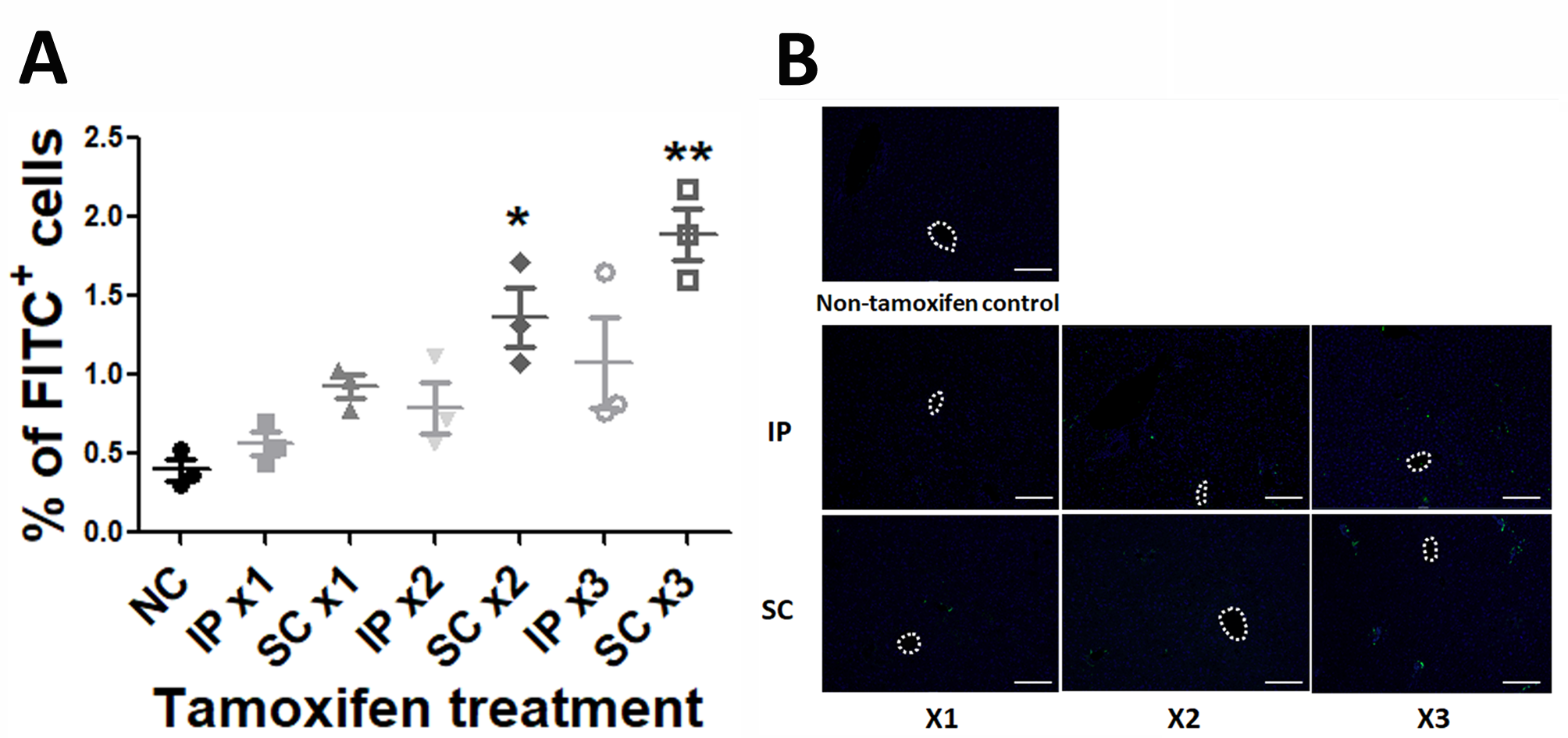

Supplement: Supplementary file 1 [file JCMM-24-1578-s001.tif]

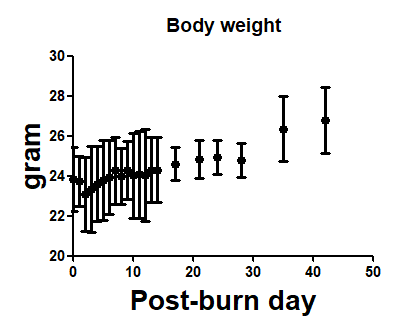

Supplement: Supplementary file 2 [file JCMM-24-1578-s002.tif]

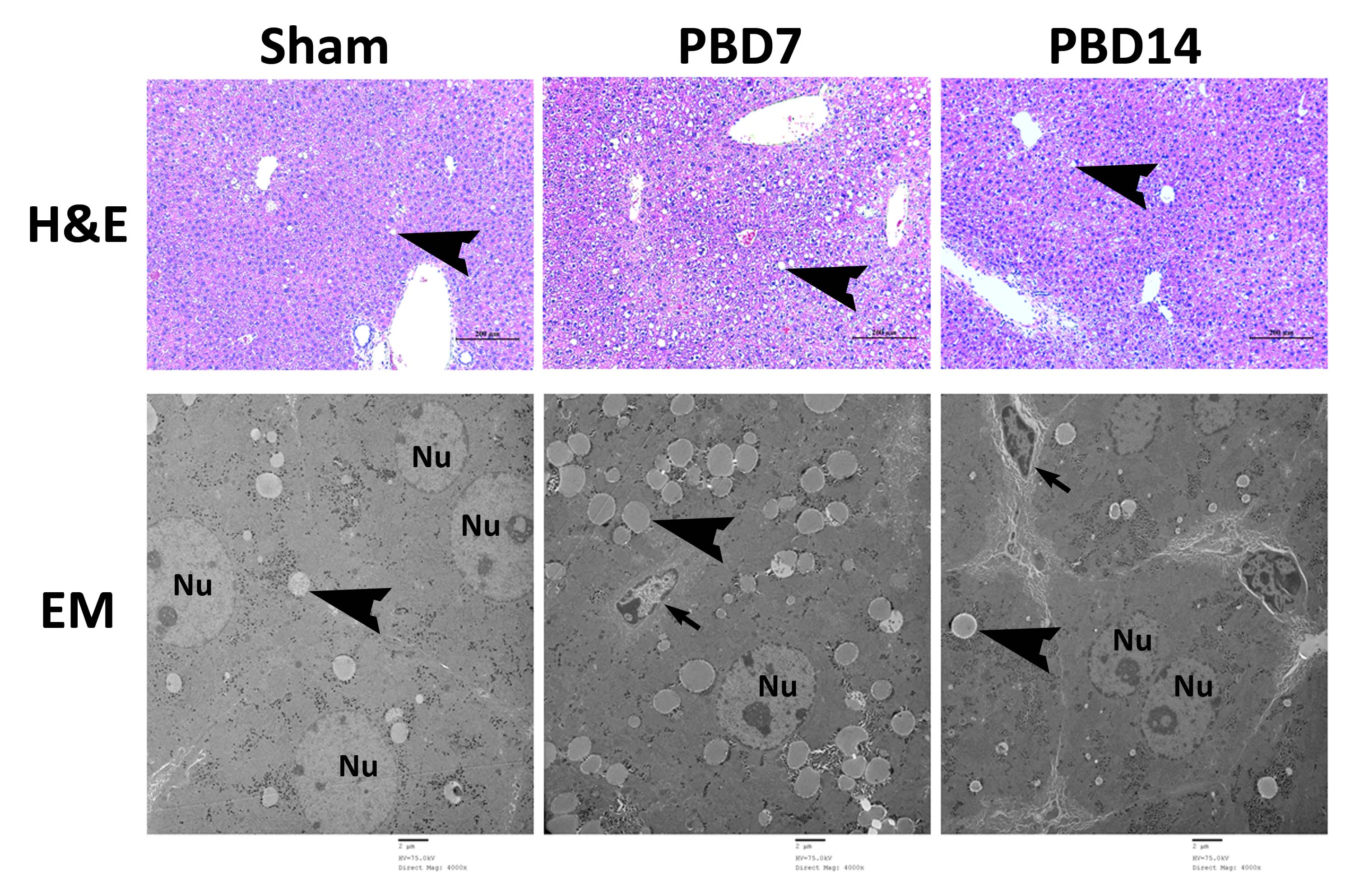

Supplement: Supplementary file 3 [file JCMM-24-1578-s003.jpg]
