# Supplementary material for: Increased proliferation of hepatic periportal ductal progenitor cells contributes to persistent hypermetabolism after trauma
Source: J Cell Mol Med. 2019 Dec 3;24(2):1578–87. doi: 10.1111/jcmm.14845 (PMC6991656; doi:10.1111/jcmm.14845)
Supplement: Supplementary file 4 [file JCMM-24-1578-s004.docx]

**Supplementary Table S1** **Primers for genotyping**

| **Target** | **Primer** | **Sequence 5' --> 3'** | **Primer Type** | **PCR product size** | | |
| --- | --- | --- | --- | --- | --- | --- |
| Sox9 Cre | oIMR1084 | GCG GTC TGG CAG TAA AAA CTA TC | Transgene | | 100bp |  |
|  | oIMR1085 | GTG AAA CAG CAT TGC TGT CAC TT | Transgene | |  |  |
|  | oIMR7338 | CTA GGC CAC AGA ATT GAA AGA TCT | Internal Positive Control Forward | | 324bp |  |
|  | oIMR7339 | GTA GGT GGA AAT TCT AGC ATC ATC C | Internal Positive Control Reverse | |  |  |
|  |  |  |  | |  |  |
| EYFP | oIMR4982 | AAG ACC GCG AAG AGT TTG TC | Mutant | | 320bp |  |
|  | oIMR8545 | AAA GTC GCT CTG AGT TGT TAT | Common (both bands: heterozygote) | |  |  |
|  | oIMR8546 | GGA GCG GGA GAA ATG GAT ATG | Wild type | | 600bp |  |

**Supplementary Table S2** **Comparison of the** **changes in canonical signaling pathways**

**in EYFP^+^ cells in mice of PBD7 versus Sham group**

| **Up-regulated signaling pathways** | **Down-regulated signaling pathways** |
| --- | --- |
| ***acute phase response signaling*** | ***LXR/RXR activation*** |
| coagulation system | production of NO and ROS in macrophages |
| type 1 diabetes mellitus signaling | role of NFAT in regulation of immune response |
| ***IL-6 signaling*** | B cell receptor signaling |
| ***p38 MAPK signaling*** | PI3K signaling in B lymphocytes |
|  | Th1 pathway |
|  | role of pattern recognition receptors in recognition of bacterial and virus |
|  | FcyRIIb signaling in B lymphocytes |
|  | calcium-induced T lymphocytes apoptosis |
|  | phospholipase C signaling |
|  | p70S6K signaling |

**Supplementary Table S3 Comparison of the changes in canonical signaling pathways**

**in EYFP^+^ versus EYFP^-^ cells in mice of PBD7 group**

| **Up-regulated signaling pathways** | **Down-regulated signaling pathways** | |
| --- | --- | --- |
| IL-8 signaling | ***LXR/RXR activation*** | |
| TREM1 signaling | role of NFAT in regulation of immune response | |
| Pattern recognition receptors in recognition of bacterial and virus | Th1 pathway | |
| NF-κB signaling | Th2 pathway | |
| Toll-like receptor signaling | phospholipase C signaling | |
| Type 1 diabetes mellitus signaling | PPAR signaling | |
| ***Acute phase response signaling*** | EIF2 signaling | |
| Coagulation system | Telomerase signaling | |
| ***IL-6 signaling*** | IL-2 signaling | |
| Cholecystokinin/Gastrin-mediated Signaling Pathway | PTEN signaling | |
| Cytotoxic lymphocyte mediated apoptosis | PI3K/AKT signaling | |
| HMGB1 signaling | Unfolded protein response | |
| ***P38 MAPK signaling*** |  |  |
| Tec kinase signaling |  |  |
| Integrin signaling |  |  |
| Autophagy |  |  |
| HGF signaling |  |  |
| MEF2 mediated oxidative stress responses |  |  |
| PEDF signaling |  |  |
| inflammasome pathway |  |  |
| LPS/IL-1 mediated inhibition of RXR function |  |  |
| Apoptosis signaling |  |  |
| Fas signaling |  |  |
| LPS-activated MAPK signaling |  |  |
| GM-CSF signaling |  |  |
| VEGF signaling |  |  |
| Stat3 pathway |  |  |
| NOS signaling |  |  |
| Cdc-42 signaling |  |  |
| ILK signaling |  |  |
| p53 signaling |  |  |
| Death receptor signaling |  |  |
| endothelin 1 signaling |  |  |
| CXCR4 signaling |  |  |
| phospholipase C signaling |  |  |
| p70S6K signaling |  |  |
| mTOR signaling |  |  |
| VDR/RXR activation |  |  |
| notch signaling |  |  |
| cAMP mediated signaling |  |  |
| TGF-beta signaling |  |  |
| IL-10 signaling |  |  |
| IL-22 signaling |  |  |
| phagosome formation |  |  |
| CD40 signaling |  |  |
| SAPK/ERK signaling |  |  |
| JAK/Stat signaling |  |  |
| UVB-induced MAPK signaling |  |  |
| ERK/MAPK signaling |  |  |
| VDR/RXR activation signaling |  |  |
| CNTF signaling |  |  |
| ErbB2-ErbB3 signaling |  |  |
| TNFR1 signaling |  |  |
|  | |  |
