# Supplementary material for: Increased proliferation of hepatic periportal ductal progenitor cells contributes to persistent hypermetabolism after trauma
Source: J Cell Mol Med. 2019 Dec 3;24(2):1578–87. doi: 10.1111/jcmm.14845 (PMC6991656; doi:10.1111/jcmm.14845)
Supplement: Supplementary file 5 [file JCMM-24-1578-s005.docx]

**Supplementary Fig. S1 Comparison of the induction of EYFP expression in the liver of Sox9 cre/ERT2^+/-^:ROSA26 EYFP^+/+^ mice between peritoneal or subcutaneous injection of tamoxifen by flow cytometry and immunofluorescent staining of fluorescent protein.**

**A** EYFP^+^ cell counts from liver of tamoxifen treated mice by flow cytometry.

**B** Representative images of immunofluorescent staining of fluorescent protein GFP in the liver tissue section of mice treated with tamoxifen. Tamoxifen was injected once daily. NC: non-tamoxifen control; IP: intraperitoneal injection; SC: subcutaneous injection. Data are presented as means ± SEM. **p*<0.05 and ***p*<0.01 as compared with non-tamoxifen control group. N=3 animals per group including non-tamoxifen control and different time point post-burn. Scale bar = 100μm.

**Supplementary Fig. S2 The changes in body weight of the mice were not significant during the time course study.** Data are presented as means ± SEM. The number of the burned and sham-burned mice decreased along the time course study from 36 at the beginning of the experiment to 6 by post-burn day 42, respectively.

**Supplementary Fig. S3 Hepatic fat infiltration in early post-burn period.** Representative images of H&E staining (×100) and electron microscope (×4000) of the liver tissue section of sham and burned mice. Nu: nucleus. Arrowheads: lipid droplets. Arrows: infiltrated monocytes. PBD: post-burn day.
